# Dataset of Bessel function $J_{n}$ maxima and minima to 600 orders and 10000 extrema

**DOI:** 10.1016/j.dib.2021.107508

**Published:** 2021-10-30

**Authors:** Nicholas A. Mecholsky, Sepideh Akhbarifar, Werner Lutze, Marek Brandys, Ian L. Pegg

**Affiliations:** a Vitreous State Laboratory, United States; b Department of Physics, The Catholic University of America, 620 Michigan Ave NE, Washington, D.C. 20064, United States

**Keywords:** Bessel functions, GCF, Extrema, Minimum, Maximum

## Abstract

Bessel functions of the first kind are ubiquitous in the sciences and engineering in solutions to cylindrical problems including electrostatics, heat flow, and the Schrödinger equation. The roots of the Bessel functions are often quoted and calculated, but the maxima and minima for each Bessel function, used to match Neumann boundary conditions, have not had the same treatment. Here we compute 10000 extrema for the first 600 orders of the Bessel function J. To do this, we employ an adaptive root solver bounded by the roots of the Bessel function and solve to an accuracy of $10^{-19}$. We compare with the existing literature (to 30 orders and 5 maxima and minima) and the results match exactly. It is hoped that these data provide values needed for orthogonal function expansions and numerical expressions including the calculation of geometric correction factors in the measurement of resistivity of materials, as is done in the original paper using these data.

## Specifications Table

**Table:** 

Subject	Applied Mathematics
Specific subject area	Locations of the maxima and minima of the Bessel function of the First Kind, $J_{n}\left(r\right)$. We refer to these extrema as $e_{n,m}$.
Type of data	Table
How data were acquired	Wolfram Mathematica 12.0.0 for Linux x86 (64 bit)
Data format	Raw
Parameters for data collection	Each extrema (maximum or minimum) for the Bessel function $J_{n}\left(r\right)$ starting from r=0 counting from the first maximum (m=1) to the 10000th extrema (m=10000). Here, n is the order of the Bessel function in the range 0≤n≤600.
Description of data collection	These extrema are calculated using an adaptive root solver bounded by the roots of the Bessel function $J_{n}\left(r\right)$.
Data source location	Institution: The Catholic University of America City/Town/Region: Washington, D.C. Country:USA
Data accessibility	Repository name: Mendeley Data, V1 Data identification number: https://doi.org/10.17632/cdhw4wn5sy.1 Direct URL to data: https://data.mendeley.com/datasets/cdhw4wn5sy/1
Related research article	[1] S. Akhbarifar, N. A. Mecholsky, M. Brandys, W. Lutze, and I. L. Pegg, Four-point probe geometric correction factor for isotropic cylindrical samples with non-equal probe distances, *Measurement* 184 (2021): 109703. https://doi.org/10.1016/j.measurement.2021.109703

## Value of the Data

• These calculated extrema will provide access to important physical properties of the Bessel function of the First Kind, $J_{n}$. The Bessel function zeros have been tabulated for many years, but an extensive list of the maxima and minima is lacking.
• Anyone in the sciences and engineering seeking numerical solutions to cylindrical problems including problems in electrostatics, heat flow, and the Schrödinger equation will be able to use these data.
• These data may be used to check behaviors of the roots and extremal points. Asymptotic expansions and other approximations may be tested as well.

## 1 Data Description

The data are in a Tab Separated Value (TSV) format with no headers. Each row is a separate Bessel function order starting from 0, and ending at 600. Each row contains 10,000 tab separated text values with a fixed 19 places to the right of the decimal point.

## 2 Experimental Design, Materials and Methods

**Fig. 1:**
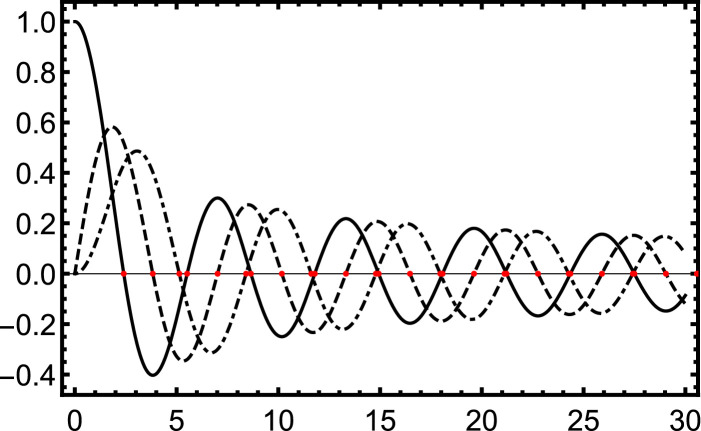
The first 3 Bessel functions. Solid is $J_{0}$, dashed is $J_{1}$, and dot dashed is $J_{2}$. The red dots indicate the zeros of the Bessel functions. Each maxima and minima is bounded by the zeros of the Bessel function and thus provide a simple way to find each root of the derivative of the Bessel function.

**Fig. 2:**
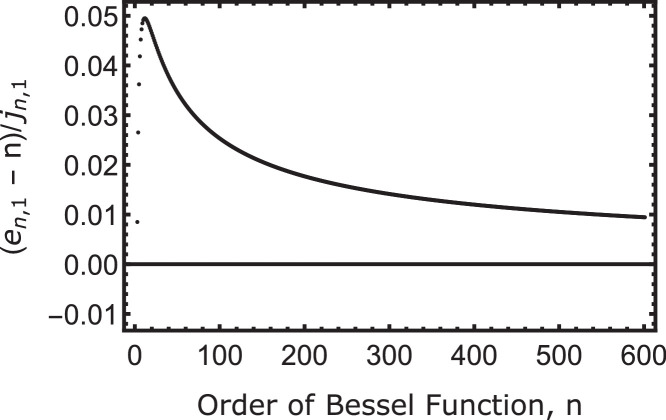
Location of first maximum minus the order number divided by the first zero of the nth Bessel function versus the order number. This shows that the order number, n is a good approximate starting location for the first maximum considering that the first Bessel zero (i.e., the size of the range wherein the first maximum occurs) goes like the order.

The Bessel functions of the first kind with nonnegative order (for example see Fig. 1) all have the same basic shape [5], [6]. Additionally, the zeros of the Bessel J are widely used and incorporated into most programming languages, including Mathematica. The maxima and minima of each Bessel $J_{n}$, here referred to as $e_{n,m}$, are the zeros of the derivative of the Bessel functions with respect to their argument and are bounded by the Bessel zeros. Therefore we can use the tabulated zeros to provide a bound to numerically solve for any extremum (see Fig. 1). For instance in Fig. 1, the third extremum (a maximum) of $J_{1}$, $e_{1,3}$, is between the second zero, $j_{1,2}$, and the third zero, $j_{1,3}$. The only difficulty is the first maximum for all n, especially when n gets larger. Since the first zero goes as $j_{n,1}∼n+1.86n^{1/3}$ where $j_{n,m}$ is the mth zero of the nth Bessel J (see Equation 10.21.40 of Ref. [3], and Refs. [4], [6]) the range over which to search for a maximum gets larger and larger. For only this first extremum point, it is helpful to use a starting value, n, where n is the order of the Bessel function as an initial guess to start the numerical root solving. This is the lowest order approximation for the zero of the Bessel function as given in Equation 10.21.40 of Ref. [3]. As can be seen in Fig. 2, this is a pretty good approximation for the first extremum at least for the first 600 Bessel functions.

The derivative of the Bessel function is given by the recursion relation (see Ref. [3], 10.6.2 and Refs. [2], [6])

(1) $$J_{n}^{\prime }\left(z\right)=\frac{1}{2}\left(J_{n-1}\left(z\right)-J_{n+1}\left(z\right)\right).$$

The roots of this expression are then found using standard built in root finding methods of Mathematica to an accuracy of $10^{-19}$.

The function to accomplish this in Mathematica is:



To test the accuracy of these solutions, we evaluate each extremum into the derivative of the Bessel function, Eqn. (1), to determine if it is a root. Evaluating every extremum, $J_{n}^{\prime }\left(e_{n,m}\right)$, the error is not more than the stated accuracy for every entry ($10^{-19}$).

## CRediT Author Statement

**Nicholas A. Mecholsky:** Conceptualization, Investigation, Methodology, Formal analysis, Software, Validation, Visualization, Data curation, Writing – original draft, Funding acquisition; **Sepideh Akhbarifar:** Investigation; Project administration; Writing – review & editing; **Ian L. Pegg:** Funding acquisition.

## Declaration of Competing Interest

The authors declare that they have no known competing financial interests or personal relationships which have, or could be perceived to have, influenced the work reported in this article.

## Appendix A Supplementary materials

Supplementary Data S1 Supplementary Raw Research Data. This is open data under the CC BY license http://creativecommons.org/licenses/by/4.0/ Supplementary Data S1
